# Pituitary Tumors: Molecular Insights, Diagnosis, and Targeted Therapy

**DOI:** 10.3390/cancers15235526

**Published:** 2023-11-22

**Authors:** Kazunori Kageyama, Mitsuru Nishiyama

**Affiliations:** 1 Department of Endocrinology and Metabolism, Hirosaki University Graduate School of Medicine, 5 Zaifu-cho, Aomori 036-8562, Japan; 2 Health Care Center, Kochi University, 1-5-2 Akebono-cho, Kochi 780-8520, Japan; nisiyamm@kochi-u.ac.jp

The anterior pituitary gland comprises a heterogeneous population of pituitary cells. Anterior cells are mainly characterized as corticotrophs, somatotrophs, lactotrophs, thyrotrophs, and gonadotrophs. Pituitary stem cells can differentiate into five hormone-producing cell types. This differentiation requires a complex cascade and is driven by specific transcription factors during development. Pituitary neuroendocrine tumors (PitNETs), formerly known as pituitary adenomas, are common intracranial tumors originating from the anterior pituitary neuroendocrine cells [1]. PitNETs are defined as autonomous/dysregulated cell proliferations or secretions of pituitary hormones. They present with various hormonal activities and clinical features, ranging from overt to subtle. In this Special Issue, we explore recent advances in molecular insights, diagnosis, and targeted therapy for PitNETs. The outlined papers include original clinical research articles and reviews on the pathophysiology, diagnosis, and potential treatment of PitNETs.

Oh et al. addressed the PitNET pathogenesis [2]. PitNETs are classified according to their lineage-restrictive pituitary transcription factors (*TPIT* for corticotroph tumors; *PIT1* for somatotroph, lactotroph, and thyrotroph tumors; *SF1* for gonadotroph tumors; the absence of *PIT1*, *TPIT*, and *SF1* for null cell tumors). Less than 5% of PitNETs develop from germline mutations as a part of syndromic diseases or as a familial isolated pituitary adenoma [3], whereas the remaining 95% develop in the context of sporadic, somatic mutations in various genes that regulate the cell cycle, cell signaling, and transcriptional changes [4], lacking ubiquitous changes. Unlike hypersecreting PitNETs, nonfunctioning PitNETs may be found incidentally or with signs of mass effects rather than symptoms of excessive hormone secretion. Some nonfunctioning PitNETs are silent and show normal hormone secretion and anterior hormone or transcriptional factor-immunopositive staining without autonomous hormone secretion. Most silent PitNETs are silent gonadotrophs and all hypersecreting PitNET types have silent counterparts, such as silent lactotrophs, somatotrophs, and corticotroph tumors.

Tahara et al. reviewed pituitary incidentalomas [5]. The most common pituitary incidentalomas are PitNETs and Rathke cleft cysts. Surgical resection is recommended in cases of clinically nonfunctioning PitNETs with optic chiasm compression, whereas cystic lesions, such as Rathke cleft cysts, are followed up if the patients are asymptomatic. In contrast, certain pathological types of PitNETs, such as immature pluripotent PitNET lineage *PIT1*, Crooke’s cell tumor variant corticotroph PitNETs, and silent corticotroph PitNETs, are aggressive and therefore require careful monitoring.

Cushing’s disease is defined as the autonomous excess adrenocorticotropin (ACTH) secretion in corticotroph tumors (*TPIT*-lineage PitNETs) and excess cortisol production, with the clear manifestation of the clinical features of Cushing’s disease [6]. Mutations in the *ubiquitin-specific protease* (*USP*) *8* genes have been detected in Cushing’s disease [7,8,9]. This mutation increases enzyme activity, resulting in the excessive deubiquitination of epidermal growth factor receptor (EGFR) tyrosine kinase, disturbing its degradation [7]. EGFR expression levels positively correlate with ACTH production and cell proliferation in corticotrophic PitNETs. Phosphorylated EGFR expression has been reported in most corticotroph PitNETs [10], and an EGFR inhibitor could be effective in treating EGFR-related tumors. Gefitinib, a known EGFR tyrosine kinase inhibitor, has been found to suppress ACTH production and tumor growth in an experimental mouse model of Cushing’s disease using AtT-20 allografts [11]. Lapatinib, an active specific tyrosine kinase receptor inhibitor of both EGFR and p185her2/neu (HER2), significantly decreases ACTH production and corticotroph tumor cell proliferation [12]. 

A somatostatin analog with preferential affinity for somatostatin receptor type 5 (SSTR5) inhibits ACTH secretion in patients with ACTH-secreting PitNETs [13]. SSTR-mediated inhibition of phosphorylated extracellular signal-related kinases (ERK) via pasireotide decreases corticotrophic tumor cell proliferation [14]. Pasireotide may induce tumor shrinkage by directly acting on tumor cells [15]. Recently, researchers have found that SSTR5 is highly expressed in *USP8*-mutated tumors and pasireotide reportedly increases its antisecretory response in these tumors [16]. The overexpression of heat shock protein 90 (HSP90) has been reported in Cushing’s disease [17], which restrains the release of mature glucocorticoid receptors and causes partial glucocorticoid resistance. Silibinin, a C-terminal HSP90 inhibitor, restores glucocorticoid sensitivity [17]; therefore, an appropriate HSP90 inhibitor could be an effective treatment [18]. Some histone deacetylase inhibitors have been shown to block cell proliferation and ACTH synthesis in mouse AtT-20 and human corticotroph PitNETs [19,20,21,22,23]. The overexpression of the cell cycle regulator cyclin E and low expression of the cell cycle inhibitor tumor protein 27Kip1 (p27) have been observed in Cushing’s disease [24,25]. Cyclin E expression is correlated with p27 loss in human corticotroph PitNETs [26]. R-roscovitine, a pharmacological cyclin-dependent kinase 2 (CDK2)/cyclin E inhibitor, has also been shown to inhibit tumor growth in a mouse model of corticotroph tumors [27,28]. Overall, developments involving HSP90, histone deacetylase, and cyclin-dependent kinase inhibitors are expected for the treatment of Cushing’s disease [6]. 

In acromegaly, various genetic and epigenetic factors are involved in the somatotroph PitNET pathogenesis [29]. Somatic mutations of *GNAS* (*Guanine nucleotide activating subunit*) are the most prevalent cause of somatotroph PitNETs, whereas germline mutations in various genes, such as *Aryl hydrocarbon receptor-interacting protein* (*AIP*), *Protein kinase cAMP-dependent type 1 regulatory subunit alpha* (*PRKAR1A*), *G protein-coupled receptor 101* (*GPR101*), *GNAS*, *MEN1* (*MENIN*), *Cyclin-dependent kinase inhibitor 1B* (*CDKN1B)*, *Succinate dehydrogenase* (*SDHx*), and *MYC associated factor X* (*MAX*), also cause somatotroph PitNETs. DNA hypomethylation and higher mRNA expression of *potassium voltage-gated channel subfamily A regulatory beta subunit 2* (*KCNAB2*) have been observed in somatotrophs than in nonfunctioning pituitary PitNETs [30,31]. The pathophysiological relevance of miRNAs in somatotroph PitNETs has also been reported [29]. 

The entirety of the underlying mechanisms of somatotroph PitNETs has been clarified by multiple perspectives of the pan-genomic approach, including genome, transcriptome, methylome analyses, histological characterization, genomic instability, and possible miRNA involvement. Ferrés et al. determined the prognostic variables that facilitated predicting long-term postoperative outcomes to guide conceiving the most accurate acromegaly management practices [32]: younger age, higher preoperative growth hormone (GH), and insulin-like growth factor-1 levels, group 2b clinicopathological classification, Knosp’s grade IV, and magnetic resonance imaging. T2-weighted tumor hyperintensity and sparsely granulated cytokeratin expression patterns are associated with inferior postoperative outcomes in the long-term follow-up of patients with GH-secreting PitNETs. 

Fukuhara et al. [33] updated the pathogenesis, diagnosis, and treatment of prolactinomas. Prolactinomas, comprising 30–50% of all PitNETs, are divided into two subgroups: familial and sporadic. Prolactinoma is the most frequent familial PitNET in patients with multiple endocrine neoplasia type 1 (MEN1) [34], whereas *MEN1* gene mutations are also found in some sporadic prolactinomas. In addition, prolactinoma has been found in families with mutations in the *PRKAR1A* (Carney complex), *CDKN1B* (MEN4), or *AIP* (familial isolated pituitary adenoma: FIPA) genes [35]. Most prolactinomas can be treated using dopamine agonists. Temozolomide may be effective against malignant prolactinomas and a good response is obtained when O6-methylguanine-DNA methyltransferase immunostaining is negative. New medical treatments using estrogen receptor (ER) antagonists such as tamoxifen or raloxifene have also shown promising results [36] because ER is expressed in most surgical prolactinoma specimens. Recently, mammalian targets of rapamycin or tyrosine kinase inhibitors have been reported to be effective against aggressive prolactinomas [37,38]. 

Matsumoto et al. developed pituitary induction methods using human-induced pluripotent stem cells (hiPSCs), advancing the induction of pituitary glands from mouse embryonic stem cells [39,40]. Genome editing technologies using CRISPR/Cas systems have become standard methods. Additionally, the CRISPR/Cas system can introduce causative mutations from various malignant tumors into hPSCs. Therefore, combining hPSC-derived pituitary hormone-producing cells and genome-editing technologies may be useful for pituitary tumor research and developing novel drugs in the future. 

## Data Availability

Not applicable.
